# Humans with obesity exhibit impaired circulating total, but not free, IGF‐1 response to acute endurance exercise

**DOI:** 10.14814/phy2.70436

**Published:** 2025-06-26

**Authors:** Eduardo D. S. Freitas, Lori R. Roust, Eleanna De Filippis, Matthew Buras, Brooke Brown, Christos S. Katsanos

**Affiliations:** ^1^ School of Life Sciences Arizona State University Tempe Arizona USA; ^2^ College of Medicine Mayo Clinic Arizona Scottsdale Arizona USA; ^3^ Department of Biomedical Informatics Mayo Clinic Arizona Scottsdale Arizona USA; ^4^ Clinical Studies Unit Mayo Clinic Arizona Scottsdale Arizona USA; ^5^ Department of Physiology and Biomedical Engineering Mayo Clinic in Arizona Scottsdale Arizona USA

**Keywords:** exercise, growth hormone, IGF‐1, IGFBP‐1, IGFBP‐3, obesity

## Abstract

We investigated how obesity impacts the response of circulating insulin‐like growth factor‐1 (IGF‐1) to a single bout of endurance exercise in humans with and without obesity. Blood samples were collected before exercise, at 15 and 40 min during a 45‐min cycling session at 65% of heart rate reserve, and 15 min post‐exercise. Serum levels of total IGF‐1, free IGF‐1, IGF binding proteins 1 (IGFBP‐1) and 3 (IGFBP‐3), growth hormone, and insulin were measured. Both total IGF‐1 and IGFBP‐3 serum concentrations increased significantly (*p* < 0.05) during exercise in the study participants without obesity but not in those with obesity, returning to basal levels immediately after exercise. There was a statistically significant main effect on the growth hormone response, with circulating levels being higher in participants without obesity (*p* < 0.05). No significant effects were observed for either free IGF‐1 or IGFBP‐1 serum concentrations in response to exercise in either group (*p* > 0.05). We conclude that humans with obesity have blunted serum total IGF‐1 response during exercise. However, a concurrent attenuation in serum IGFBP‐3 response, which regulates free (i.e., biologically active) IGF‐1 in circulation, results in no change in circulating free IGF‐1 levels during endurance exercise in individuals with obesity.

## Graphical abstract

Circulating total IGF‐1 response to acute endurance exercise is impaired in obesity.

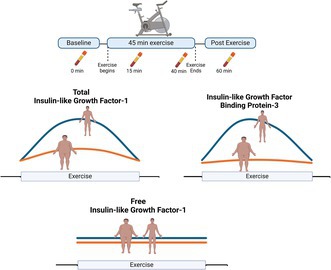

## 1 INTRODUCTION

Obesity is a major global public health issue, affecting over 40% of US adults, with significant associated health burdens (Stierman & Carroll 2024). Obesity is increasingly acknowledged as a condition that profoundly disrupts the endocrine system, resulting in various hormonal imbalances (Ylli et al., 2000). The hormone insulin‐like growth factor‐1 (IGF‐1), synthesized primarily in the liver and secreted into the circulation (Adamo et al., 1993), is directly involved in cell differentiation, maturation, growth, and proliferation across nearly all body tissues (Bailes & Soloviev, 2021; Jones & Clemmons, 1995). It also plays a key role in protein, carbohydrate, and lipid metabolism (Kineman et al., 2018; Yoshida & Delafontaine, 2020), exerts antioxidant and anti‐inflammatory effects on the vasculature (Higashi et al., 2014), and is crucial for regulating cardiac function (Macvanin et al., 2023). Research has shown that individuals with obesity exhibit either normal or low serum IGF‐1 levels in the fasting state (Clasey et al., 2001; Gram et al., 2006; Juiz‐Valina et al., 2020; Juul, 2003; Lukanova et al., 2004; Tran et al., 2018). Understanding how circulating IGF‐1 concentrations are regulated, not only during fasting but also under various physiological conditions, is important for mitigating risks associated with its dysregulation.

Acute exercise increases immediately (Cappon et al., 1985) circulating levels of IGF‐1 in healthy humans (Berg & Bang, 2004; Cappon et al., 1985; de Alcantara Borba et al., 2020; Kelly et al., 2025; Schwarz et al., 1996). This effect is more pronounced with endurance exercise compared to resistance exercise (de Alcantara Borba et al., 2020), although making a direct comparison between the two is challenging due to difficulties in matching intensity and duration between exercise modalities (de Alcantara Borba et al., 2020). Given the multifaceted role of circulating IGF‐1 in mediating many beneficial responses to exercise, a potentially blunted response in individuals with obesity could have important implications for the effectiveness of exercise interventions, particularly considering that exercise is a cornerstone of obesity management and the prevention of related health complications. For example, the protective effects of exercise on the brain are thought to be mediated by circulating IGF‐1 levels (Carro et al., 2001), as are the acute effects of exercise on attenuating vascular dysfunction and hypertension (Bass‐Stringer et al., 2021; Norling et al., 2020).

In children with obesity, acute exercise does not impair increases in circulating IGF‐1 levels, supporting its use as a lifestyle intervention to modulate IGF‐1 in this population (Eliakim et al., 2006). However, aging is a major factor that inversely affects circulating IGF‐1 levels (Goodman‐Gruen & Barrett‐Connor, 1997; Holmes et al., 2002), indicating that findings in children cannot be directly extrapolated to adults with obesity. Hepatic secretion of IGF‐1 is primarily regulated through the growth hormone‐IGF‐1 axis, in which growth hormone stimulates IGF‐1 production in the liver (Yamamoto & Bando, 2023), which leads to increased levels of both total and free (i.e., unbound or biologically active) IGF‐1 in circulation. The age‐related decline in the responsiveness of this axis is more pronounced in individuals with obesity (Gomez et al., 2004). Furthermore, the growth hormone response to exercise is reduced in older overweight adults compared to their lean counterparts (Holt et al., 2001). Thus, an attenuated circulating total and free IGF‐1 response to exercise in individuals with obesity may limit the full therapeutic potential of exercise to improve metabolic function, muscle and cardiovascular health, and cognitive performance in these individuals.

Therefore, the primary goal of this study was to compare circulating concentrations of IGF‐1 and its binding proteins at rest, during an acute bout of endurance exercise, and immediately afterward in adults with and without obesity. We hypothesized that individuals with obesity would exhibit an attenuated increase in circulating IGF‐1 levels in response to acute endurance exercise, with levels returning to baseline immediately after the cessation of exercise.

## 2 MATERIALS AND METHODS

### 2.1 Study participants

The study included individuals with obesity and a control group of individuals without obesity (Table 1). The inclusion criteria consisted of Body Mass Index (BMI) between 18 and 25 kg/m^2^ (i.e., individuals without obesity) and 32 and 50 kg/m^2^ (i.e., individuals with obesity). Study participants were classified as physically inactive based on current guidelines, defining inactivity as engaging in regular physical activity for two or fewer days per week (Piercy et al., 2018; Tremblay et al., 2017). Preliminary studies (i.e., 4 participants per group) indicated an effect size (f) of 0.30 for the difference in total IGF‐1 response to exercise between groups. Power calculations (i.e., G*Power) based on an effect size of 0.30, an *α* level of 0.05, and a power of 0.80, indicated that a total sample size of 20 participants (10 per group) would be sufficient to detect significant interaction in a repeated measures ANOVA.

**TABLE 1:** Participant anthropometric and blood biochemical characteristics.

	Without obesity	With obesity	*p*
	(*n* = 10)	(*n* = 11)	
Sex (males/females)	7/3	7/4	‐
Race	9W/1AA	10W/1AA	‐
Age (years)	27 ± 8	29 ± 9	0.59
Weight (kg)	73 ± 11	106 ± 14	<0.001
BMI (kg/m^2^)	24 ± 2	36 ± 4	<0.001
Waist circumference (cm)	82 ± 7	118 ± 18	< 0.001
Waist‐to‐hip ratio (cm)	0.87 ± 0.08	0.99 ± 0.09	<0.01
FFM (kg)	56 ± 10	66 ± 9	<0.05
Body fat percentage (%)	22 ± 7	37 ± 7	<0.001
VO_2_peak (mL/min)	2371 ± 739	2275 ± 473	0.73
VO_2_peak (mL/kg FFM/min)	43 ± 13	35 ± 7	0.10
Glucose (mg/dL)	84 ± 5	82 ± 7	0.53
Insulin (μIU/mL)	6.7 ± 3.7	12.7 ± 6.2	0.02
Total IGF‐1 (ng/mL)	204 ± 48	166 ± 86	0.23
Free IGF‐1 (ng/mL)	4.2 ± 7.7	1.9 ± 2.9	0.37
IGFBP‐1 (ng/mL)	3.2 ± 2.3	1.7 ± 1.1	0.08
IGFBP‐3 (ng/mL)	2937 ± 1038	2696 ± 994	0.59
Growth hormone	2.2 ± 2.1	0.6 ± 1.0	0.04
Matsuda‐ISI	11.4 ± 7.4	3.7 ± 1.8	<0.01
HOMA‐IR	1.4 ± 0.8	2.6 ± 1.5	0.03

*Note*: Values are mean ± SD. Groups were compared using unpaired *t‐*test.

Abbreviations: AA, African American; BMI, Body Mass Index; FFM, fat‐free mass; HOMA‐IR, Homeostatic Model Assessment of Insulin‐Resistance; IGF‐1, insulin growth factor‐1; IGFBP‐1, IGF binding protein‐1; IGFBP‐3, IGF binding protein‐3; Matsuda‐ISI, Matsuda Insulin‐sensitivity Index; VO_2_peak, peak oxygen uptake; W, white.

The purpose of the study, the experimental procedures, and associated risks were reviewed with each participant prior to obtaining written consent. Screening procedures included medical history, standard physical examination, electrocardiogram, routine blood tests, and urinalysis. Interested participants were excluded from the study if there was evidence of acute illness, had a history or evidence based on the screening procedures of diabetes, heart, liver, kidney, or gastrointestinal diseases. None of the study participants were actively trying to lose weight. The study was approved by the Institutional Review Board at Mayo Clinic.

### 2.2 Study design

The overall study procedures involved a total of three visits to the Ambulatory Infusion Center at Mayo Clinic in Scottsdale, Arizona, with study participants arriving between 7:00 and 8:00 am. Prior to their visits participants fasted for at least 10 h. During their first visit, they underwent a physical examination and provided medical history information. Following that, those who qualified for the study completed an oral glucose tolerance test (OGTT). Plasma glucose and insulin responses during the OGTT were used to estimate the insulin sensitivity of the study participants by calculating the Matsuda Insulin Sensitivity Index (Matsuda‐ISI) (Matsuda & DeFronzo, 1999). Participants were excluded if their plasma glucose was >126 mg/dL before the OGTT or >200 mg/dL at any time point during the OGTT (i.e., individuals with diabetes). Waist and hip circumferences were measured on the same day for each participant using standardized procedures to calculate the waist‐to‐hip ratio (WHO, 2011).

In a follow up visit, participant's body composition was determined early in the morning using Bioelectrical Impedance Analysis (BIA 310e, Biodynamics Corp., Shoreline, WA). Participants were asked to abstain from food and fluid intake for at least 10 h to minimize effects of recent food consumption and acute hydration on body composition estimates (Jeong et al., 2023). Following the body composition determination, peak oxygen consumption (VO_2_peak) was measured during exercise on a cycle ergometer using a metabolic cart (MedGraphics Metabolic Cart, Saint Paul, MN). For the VO_2_peak test, after a 5‐min warm‐up, the work rate on the cycle ergometer increased by 20 watts/min, while participants maintained a pedaling rate of 65 revolutions/min. Study participants were verbally encouraged during the test to perform to exhaustion. During their third visit, participants arrived in a fasted state in the morning to complete an acute bout of endurance exercise. Blood samples were collected before, during, and after the exercise session, and participants remained fasted throughout these periods. The exercise consisted of 45 min of cycling at 65% of the participant's heart rate reserve, which was calculated based on their peak heart rate during the VO_2_peak test. The participant's heart rate was continuously monitored during exercise, and the workload was adjusted as needed to maintain heart rate within 5 beats per min of the target. Blood samples were obtained from an antecubital vein via an IV line placed prior to exercise and maintained with a saline drip throughout the study. Samples were collected immediately before exercise, at 15 and 40 min during exercise, and 15 min after completion of the exercise (i.e., 60 min from the start of exercise). Samples were collected in pre‐chilled Vacutainer® SST™ tubes (i.e., serum measurements) and sodium fluoride/potassium oxalate tubes (i.e., plasma glucose measurement). All tubes were kept on ice until centrifugation for serum or plasma separation.

### 2.3 Immunosorbent assays and other biochemical procedures

Enzyme‐linked immunosorbent assays (ELISAs) were used to measure serum concentrations of the parameters of interest, with intra‐assay and inter‐assay coefficient of variation, respectively, provided in parentheses: total IGF‐1 (ALPCO, catalog number 22‐IGFHU‐E01) (4.3%, 6.9%), free IGF‐1 (abcam, catalog number ab108873) (3.8%, 8.4%), IGF binding protein‐3 (IGFBP‐3; ALPCO, catalog number 22‐BP3HU‐E01) (3.5%, 5.4%), IGFBP‐1 (ALPCO, catalog number 11‐IGFHU‐E01) (3.2%, 6.3%), insulin (ALPCO, catalog number 80‐INSHU‐E01.1) (2.6%, 4.7%), and growth hormone (ALPCO, 25‐HGHHU‐E01) (3.5%, 7.1%). Plasma glucose concentrations were determined using an automated glucose analyzer (YSI 2300) (1.9%; 3.1%).

### 2.4 Calculations and statistical analyses

The homeostatic model assessment of insulin‐resistance (HOMA‐IR) was calculated as fasting insulin (mIU/mL) × fasting blood glucose (mg/dL)/405 (Ramesh et al., 2022). The area under the curve (AUC) for the serum concentration response to exercise relative to baseline (i.e., incremental AUC) for the variables of interest was calculated using the trapezoidal rule. AUC values reflected cumulative responses from 0 to 15, 0 to 40, and 0 to 60 min from the start of exercise.

Data normality was determined using the Levene's test and supplemented by graphical information. The unpaired t‐test was used to compare differences in basal characteristics between groups. Two‐way repeated measures ANOVA was used to assess significant main effects of time and group, as well as time × group interaction for each variable of interest. The Bonferroni correction was applied to control for familywise error rate in pairwise comparisons. The Pearson product–moment correlation coefficient (*r*) was calculated to assess the association between variables of interest. Significance level was set at *p* ≤ 0.05, and all tests were two‐tailed. All data are presented as mean ± SD.

## 3 RESULTS

Study participants with obesity exhibited a higher percentage of body fat, abdominal obesity (i.e., waist‐to‐hip ratio), and insulin resistance (i.e., HOMA‐IR) (Table 1).

**FIGURE 1:**
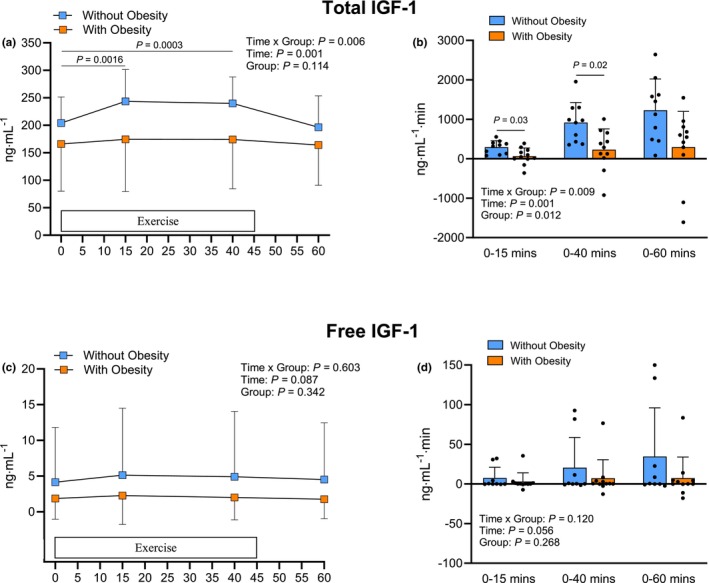
Serum total (a) and free (c) IGF‐1 responses before, during and immediately after an acute session of endurance exercise, along with the corresponding area under the curve values reflecting the cumulative responses for total (b) and free (d) IGF‐1 from 0 to 15, 0 to 40, and 0 to 60 min after the start of exercise in participants with and without obesity. Statistically significant pairwise comparisons are shown on the graphs. Values are mean ± SD.

Figure 1a shows serum total IGF‐1 concentrations in participants with and without obesity before (0 min), during (15 and 40 min), and after (60 min) the exercise session. There were significant time × group interactions (*p* < 0.01) and a time main effect (*p* < 0.001) for the serum total IGF‐1 concentrations. In participants without obesity, serum total IGF‐1 concentrations increased at both 15 and 40 min during exercise (*p* < 0.001 for both) but did not differ from pre‐exercise levels at 60 min (*p* > 0.05) (i.e., 15 min post‐exercise). However, participants with obesity showed no increase in serum total IGF‐1 concentration at any time point compared to pre‐exercise levels in response to exercise (*p* > 0.05; Figure 1a). During exercise, the AUC for serum total IGF‐1 response was 4.7‐fold higher from 0 to 15 min and 5.7‐fold higher from 0 to 40 min in participants without obesity compared to those with obesity (Figure 1b). The cumulative AUC from 0 to 60 min, which included the exercise and the 15‐min post‐exercise period, was 4.4‐fold higher in the participants without obesity but did not differ significantly between groups (*p* > 0.05). The serum free IGF‐1 concentration responses to exercise in the two groups are shown in Figure 1c. There were no significant main effects or interactions (*p* > 0.05). Contrary to the total IGF‐1 response, the concentration of free IGF‐1 in serum in participants without obesity was not significantly different from pre‐exercise levels at any time point after the start of the exercise session (*p* > 0.05), and similar findings were obtained for the participants with obesity. Similarly, no differences were detected between groups in the AUC values describing cumulative free IGF‐1 responses, as calculated using the AUC at the blood sampling time points (Figure 1d).

**FIGURE 2:**
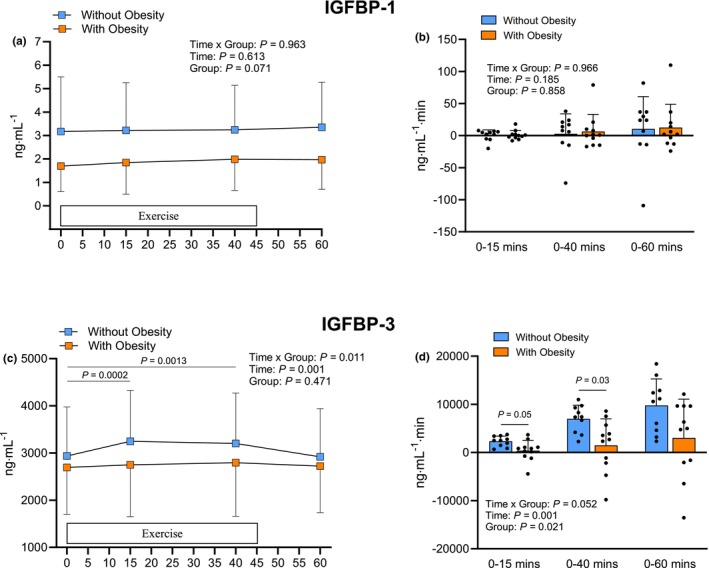
Serum IGFBP‐1 (a) and IGFBP‐3 (c) responses before, during and immediately after an acute session of endurance exercise, along with the corresponding area under the curve values reflecting the cumulative responses for IGFBP‐1 (b) and IGFBP‐3 (d) from 0 to 15, 0 to 40, and 0 to 60 min after the start of exercise in participants with and without obesity. Statistically significant pairwise comparisons are shown on the graphs. Values are mean ± SD.

The serum IGFBP‐1 concentration responses to exercise in the two groups are shown in Figure 2a. No significant main effects or interaction were found (*p* > 0.05). The concentration of IGFBP‐1 in participants without obesity was not statistically different from pre‐exercise levels at any time point after the start of the exercise session, and the same statistical findings were observed for participants with obesity (*p* > 0.05). Additionally, the calculated AUC values representing the overall serum IGFBP‐1 response over time at the blood sampling time points were not significantly different between the groups (Figure 2b). The response of IGFBP‐3 to the exercise in the two groups is shown in Figure 2c. There was a significant main effect for time (*p* < 0.001) and time × group interaction (*p* < 0.05). Serum IGFBP‐3 concentration increased during exercise in participants without obesity at both 15 (*p* < 0.001) and 40 min (*p* < 0.01) but it was not significantly different from before exercise at 60 min (Figure 2c). Participants with obesity did not display a significant increase in serum IGFBP‐3 concentration in response to exercise at any of the sampling time points. The AUC values for serum IGFBP‐3 concentration responses were 5.9‐fold higher up to 15 min (*p* = 0.05) and 4.7‐fold higher up to 40 (*p* < 0.05) during exercise in participants without obesity (Figure 2d). The AUC response over the entire 60‐min study period, which included both the exercise and a 15‐min rest period, was 3.2‐fold higher in participants without obesity, though this difference was not statistically significant between groups (*p* > 0.05).

**FIGURE 3:**
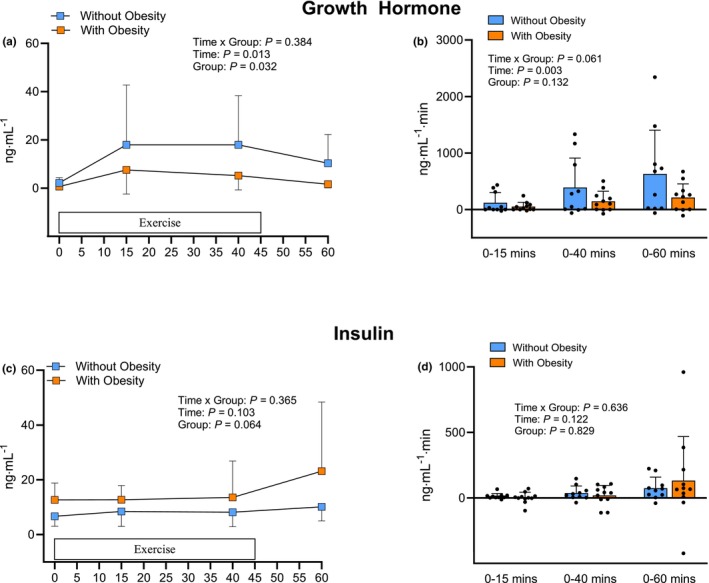
Serum growth hormone (a) and insulin (c) responses before, during, and immediately after an acute session of endurance exercise, along with the corresponding area under the curve values reflecting the cumulative responses for growth hormone (b) and insulin (d) from 0 to 15, 0 to 40, and 0 to 60 min after the start of exercise in participants with and without obesity. Values are mean ± SD.

The responses of serum growth hormone to exercise are shown in Figure 3a. These responses varied considerably across participants. There were main effects for time and group (*p* < 0.05), but no interaction (*p* > 0.05). However, pairwise comparisons showed that the increase in serum growth hormone concentration over time during exercise was not statistically significant in either group (*p* > 0.05). Despite the AUC values indicating that cumulative serum growth hormone responses to exercise were 2.2‐ and 2.6‐, and 2.9‐fold higher in the participants without obesity as assessed up to 15, 40, and 60 min after exercise, respectively, no significant differences were detected between groups (*p* > 0.05; Figure 3b). The serum insulin concentration responses in the two groups are shown in Figure 3c. There were no significant main effects or interaction (*p* > 0.05). Accordingly, no differences (*p* > 0.05) were found between groups in the AUC values, reflecting cumulative responses over time during the course of the study (Figure 3d).

Plasma glucose concentration did not differ over the course of the study in either group, nor were the cumulative responses (i.e., AUC values) different between groups (*p* > 0.05). Using the HOMA‐IR calculation, we calculated insulin resistance values based on the corresponding AUC values reflecting the cumulative insulin and glucose responses at 15, 40, and 60 min after the start of the exercise. None of these responses differed significantly between participants with and without obesity (*p* > 0.05).

**FIGURE 4:**
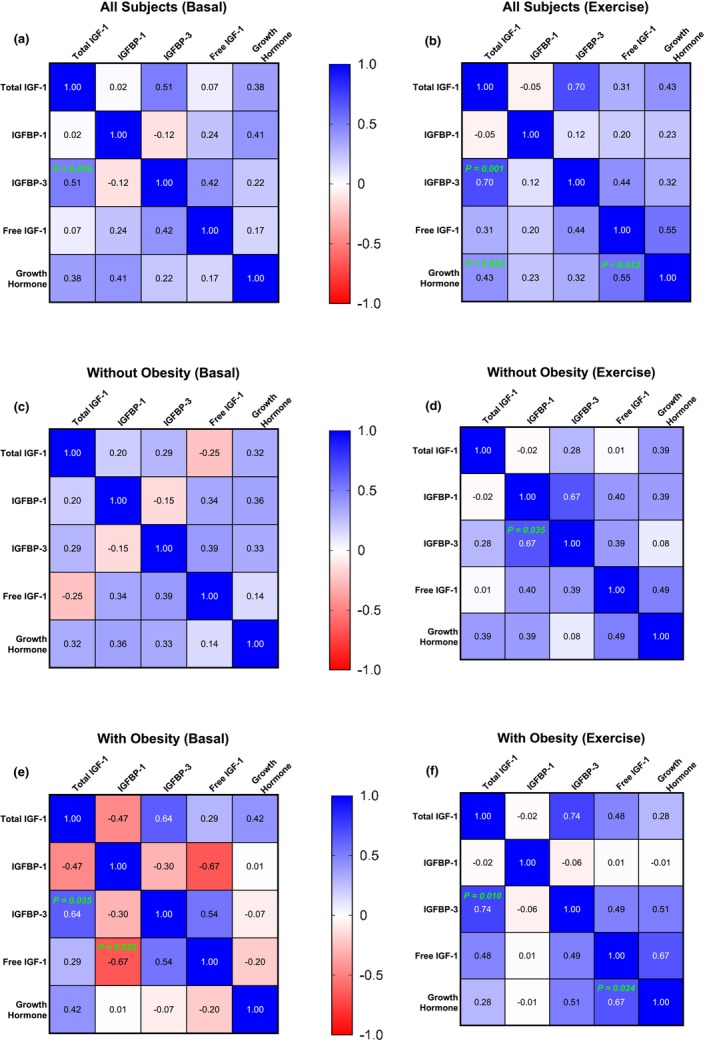
Correlation matrices visualized using heatmaps displaying Pearson's product–moment correlation coefficient (*r*) between study variables at Basal (left) and during Exercise (right) across all participants (a, b), as well as within groups of participants without (c, d) and with (e, f) obesity. Statistically significant correlations are indicated with their corresponding *p*‐values shown directly on the correlation matrices.

Across all study participants, basal serum total IGF‐1 concentrations were significantly correlated with basal serum IGFBP‐3 concentrations (*r* = 0.51; *p* < 0.05), but not with serum IGFBP‐1 or growth hormone concentrations (Figure 4a). During exercise, the correlation between serum total IGF‐1 and IGFBP‐3 concentrations became even stronger (*r* = 0.70; *p* < 0.001; Figure 4b). Also during exercise, serum total IGF‐1 and free IGF‐1 concentrations were significantly correlated with circulating growth hormone levels (*p* < 0.05). Further correlations among these variables grouped by obesity status are presented as correlation matrices in Figure 4c,d (participants without obesity) and 4E and 4F (participants with obesity). The HOMA‐IR did not significantly correlate (*p* > 0.05) with circulating total or free IGF‐1 responses, either across all participants or within the subgroups of participants with and without obesity.

## 4 DISCUSSION

We investigated the effects of acute endurance exercise on serum IGF‐1 and binding proteins of IGF‐1 in participants with and without obesity. Our key findings indicate that the exercise‐induced increase in serum total IGF‐1 is blunted in individuals with obesity. This diminished response is accompanied by a lower serum IGFBP‐3 response, highlighting a coordinated (dys) regulation of both IGF‐1 and IGFBP‐3 responses to exercise in individuals with obesity.

Similar to previous findings (Frystyk et al., 1995; Nam et al., 1997; Runchey et al., 2012), we found no significant differences in basal serum total IGF‐1 concentrations between participants with obesity and controls. Likewise, basal circulating free IGF‐1 concentrations did not differ significantly between groups, aligning with prior findings (Onder et al., 2006; Ricart & Fernandez‐Real, 2001). In agreement with evidence that acute endurance exercise increases circulating total IGF‐1 levels in healthy, lean individuals (de Alcantara Borba et al., 2020), our healthy control study participants also showed an increase in serum total IGF‐1 concentrations during exercise. Elevated serum total IGF‐1 concentrations during exercise returned to basal levels within 15 min post‐exercise, supporting previous research showing that exercise‐induced increases in circulating IGF‐1 concentrations are short‐lived (Nindl et al., 2017). However, unlike the decrease in free IGF‐1 concentrations during exercise reported in the latter study (Nindl et al., 2017), we observed no significant change from basal levels in free IGF‐1 in our healthy, control participants, a response consistent with other findings reported in healthy individuals (de Alcantara Borba et al., 2020). IGFBP‐3 is the primary binding protein for circulating IGF‐1 (Ylli et al., 2000). In our study, serum IGFBP‐3 concentrations increased during exercise in the control study participants without obesity alongside the total IGF‐1, which likely accounts for the absence of an increase in serum free IGF‐1 concentrations. This finding supports previous research showing that free IGF‐1 remains unchanged when both total IGF‐1 and IGFBP‐3 increase concurrently during exercise (Wallace et al., 1999).

We show that obesity impairs the exercise‐induced increase in serum total IGF‐1 and IGFBP‐3 concentrations. Circulating levels of IGF‐1 and IGFBP‐3 at any given time reflect a balance between their tissue production and degradation. The majority of circulating IGF‐1 is produced by liver hepatocytes (Kineman et al., 2018), a process regulated by circulating growth hormone levels (Hjelholt et al., 2020). Although pairwise comparisons in our study did not reveal any statistically significant differences, there was a significant main effect of group in the ANOVA for serum growth hormone response, with a lower response observed in participants with obesity. Therefore, it is reasonable to speculate that impaired hepatic IGF‐1 production occurred in these participants during exercise, likely due to their attenuated growth hormone response to the exercise stimulus. Interestingly, there was a trend toward a significant main effect of group on serum insulin response (*p* = 0.06), with participants with obesity exhibiting a higher response. Since hyperinsulinemia suppresses growth hormone release (Luque & Kineman, 2006), the sustained elevation in insulin levels during exercise may have contributed to the overall lower growth hormone and consequently IGF‐1 response we observed in the participants with obesity.

Despite obesity being associated with reduced basal and stimulated growth hormone secretion by the pituitary gland, circulating IGF‐1 levels are not always similarly reduced (Ylli et al., 2000). In fact, although a direct correlation between growth hormone and serum total IGF‐1 concentrations was observed during exercise across all participants (Figure 4b), this correlation was not significant within the subgroups of participants with and without obesity (Figure 4d,f), suggesting the possibility of distinct mechanisms regulating serum total IGF‐1 concentrations during exercise within each group. For example, hepatic lipid accumulation may have contributed to impaired IGF‐1 production in the participants with obesity, as circulating IGF‐1 levels are reduced in those with hepatic steatosis (Mallea‐Gil et al., 2012).

A key role of IGFBP‐3 is to regulate biologically active/free IGF‐1 in the circulation (Jogie‐Brahim et al., 2009; Yamada et al., 2010). IGFBP‐3 is primarily produced in the liver, and its expression is regulated by growth hormone (Blum et al., 1993). In response to hepatic growth hormone signaling, increased IGF‐1 secretion is accompanied by a rise in circulating IGFBP‐3 levels, which bind circulating IGF‐1 and help regulate free IGF‐1 availability (Allard & Duan, 2018). In control participants without obesity, serum total IGF‐1 and IGFBP‐3 levels increased in parallel in response to exercise, rising well above pre‐exercise values at both 15 and 40 min into the exercise session, and returning to baseline within 15 min after exercise ended. Because the production of both IGF‐1 and IGFBP‐3 occurs primarily in the liver (Allard & Duan, 2018; Blum et al., 1993), our findings suggest the presence of shared hepatic mechanisms that inhibit the secretion of both IGF‐1 and IGFBP‐3 in response to exercise in individuals with obesity. Whether this inhibition results from a lower circulating growth hormone response to exercise, since both IGF‐1 and IGFBP‐3 are regulated by growth hormone (Gu et al., 2010), or from other liver‐intrinsic factors remains to be determined. Notably, circulating IGF‐1 and IGFBP‐3 levels are both shown to be reduced in individuals with hepatic lipid accumulation (Mallea‐Gil et al., 2012; Min et al., 2016), a condition commonly observed in humans with obesity (Willis et al., 2021). Interestingly, a direct correlation was observed across all participants between serum total IGF‐1 and IGFBP‐3 concentrations at both basal and during exercise (Figure 4a,b). However, these correlations were statistically significant only within the subgroup of participants with obesity (Figure 4e,f), possibly indicating a tighter regulation of IGF‐1 and IGFBP‐3 responses in the metabolic context of obesity.

Free IGF‐1 in circulation exerts its effects on tissues by binding to its receptor on target cells (Hakuno & Takahashi, 2018). Even in the presence of comparable circulating levels of free IGF‐1, impaired tissue‐level IGF‐1 signaling can still affect IGF‐1‐regulated functions, such as glucose metabolism in muscle (Clemmons, 2006). This is particularly relevant in obesity, given our previous findings that IGF‐1 receptor expression is reduced in the muscle of humans with obesity (Freitas et al., 2023). However, we found no statistically significant differences in the estimated HOMA‐IR during the exercise session between the two groups. Moreover, the lack of significant correlations between the HOMA‐IR calculated during exercise and circulating total or free IGF‐1 responses supports previously reported findings of no correlation between IGF‐1 levels and HOMA‐IR under basal conditions (Mallea‐Gil et al., 2012) and extends this observation to the context of endurance exercise.

IGF‐1 signaling in muscle plays a key role in regulating protein metabolism in muscle (Yoshida & Delafontaine, 2020), and disruption of IGF‐1 receptor signaling impairs protein metabolism more than glucose metabolism in muscle (O'Neill et al., 2015). Because IGF‐1 is a potent stimulus for muscle protein synthesis (Bark et al., 1998; Frost & Lang, 1999), impaired IGF‐1 response may reduce protein synthesis in muscle, increasing susceptibility to muscle mass loss during periods of suboptimal dietary intake and compromising muscle quality, and because protein synthesis is essential for replacing old or damaged proteins and maintaining muscle quality (Williamson & Moore, 2021). Our findings indicate that reduced muscle protein synthesis previously reported in humans with obesity following acute exercise (Beals et al., 2018) may not be attributable to differences in the responses of circulating free IGF‐1, as its levels were comparable between participants with and without obesity in our study. However, lower basal levels of muscle‐specific IGF‐1 (Freitas et al., 2023), along with a reduced exercise‐induced increase in muscle IGF‐1 gene expression, as seen at least following resistance exercise (Sullivan et al., 2020), in humans with obesity, may contribute to impaired stimulation of muscle protein synthesis seen after exercise in individuals with obesity (Beals et al., 2018). Future research should build on our findings regarding systemic IGF‐1 responses by examining muscle‐specific IGF‐1 expression during and after endurance exercise and its relationship to muscle protein synthesis in humans with obesity. A potential limitation of our study is the lack of control for menstrual cycle phase in our female study participants. However, existing evidence indicates that the acute exercise‐induced increase in circulating IGF‐I is not affected by menstrual cycle timing (Hornum et al., 1985).

## 5 CONCLUSION

We show that humans with obesity exhibit lower circulating levels of total IGF‐1 during endurance exercise. However, this reduction is accompanied by a similarly decreased response in circulating IGFBP‐3, resulting in stable levels of free (biologically active) IGF‐1 in the circulation during exercise in humans with obesity. These findings suggest a coordinated downregulation of circulating IGF‐1 and IGFBP‐3 in response to endurance exercise, which may help maintain circulating free IGF‐1 levels in humans with obesity during exercise.

## AUTHOR CONTRIBUTIONS

Eduardo D. S. Freitas and Christos S. Katsanos conceived and designed research, interpreted results of experiments, prepared figures, drafted manuscript, and edited and revised manuscript. Eduardo D. S. Freitas, Lori R. Roust, Eleanna De Filippis, and Brooke Brown performed experiments. Eduardo D. S. Freitas, Matthew Buras, and Christos S. Katsanos analyzed data. All authors approved the final version of the manuscript.

## FUNDING INFORMATION

This work was supported by a National Institutes of Health/National Institute of Diabetes and Digestive and Kidney Diseases grant R01DK123441 (CSK).

## CONFLICT OF INTEREST STATEMENT

No competing interests, financial or otherwise, are declared by the authors.

## ETHICAL STATEMENT

Ethical approval was granted by the Institutional Review Board at Mayo Clinic

## Data Availability

The data used in this study are available upon reasonable request from the corresponding author.
